# Unusual Evolution of Plexiform Neurofibroma in the Scalp: A Case Report

**Published:** 2018-01

**Authors:** Amine Rafik, Mounia Diouri, Naima Bahechar, Abdessamad Chlihi

**Affiliations:** National Center of Burns and Plastic Surgery, University Hospital Ibn Rochd, Casablanca, Morocco

**Keywords:** Neurofibroma, Neurofibromatosis, Scalp, Schwannoma, Surgery

## Abstract

The neurofibromatosis is a frequent and polymorphic genetic disorder. The severity is related to the complications. The degeneration of neurofibroma is a very rare complication of neurofibromatosis. In the literature, a few cases of solitary neurofibroma, which turned into a malignant tumor were reported. In our case, we described a very rare clinical case of neurofibrosarcoma in the scalp, and surgical treatment.

## INTRODUCTION

Neurofibromatosis type 1 is a congenital disorder, affecting one individual in 2500 births.^1^ It is one of the most common genetic diseases, resulting due to a mutation of the NF-1 gene; The mutation is spontaneous in half of the cases and inherited as autosomal dominant in the other half.^2^ Penetrance is 100% at the age of 5 years, however expressiveness, whether for the severity or location, is extremely variable even within families whose members have inherited the same mutation.^3^

Neurofibromatosis is characterized by café-au-lait spots, lentigines and neurofibromas; with eye signs like Iris hamartoma; also CNS glioma and bone signs like dysplasia, pseudoarthrosis and scoliosis.^4^ The severity of disease usually is due to its complications. One of the more morbid complications is malignant degeneration of plexiform neurofibromas.^5^ The malignant Schwannoma is one of the forms of degeneration of the neurofibroma in neurofibrosarcoma.^6^ Here, we described a very rare clinical case of neurofibrosarcoma in the scalp, and surgical treatment.

## CASE REPORT

**Fig. 1:**
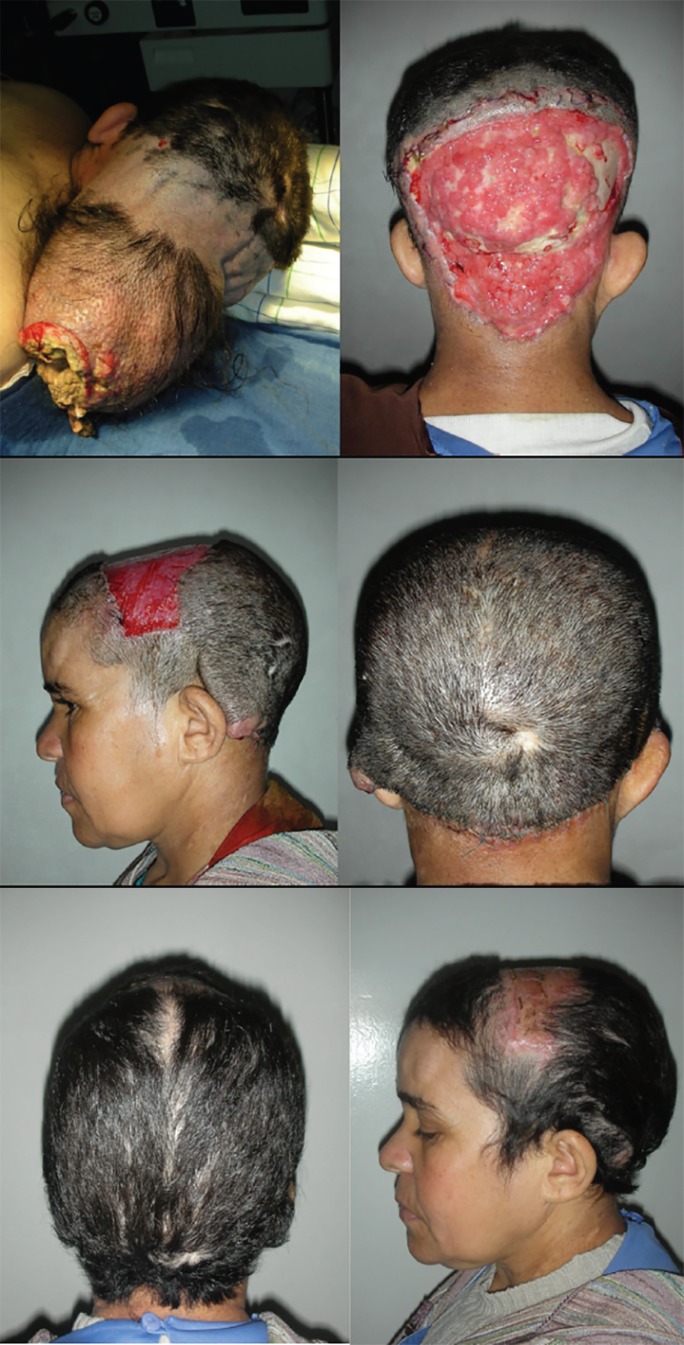
A forty-three years old patient, known carrier of Neurofibromatosis type 1 with a large occipital tumor and malignant Schwannoma.

We report our experience with a forty-three years old patient, known carrier of Neurofibromatosis type 1, who presented to us with a large occipital tumor. Biopsy revealed presence of malignant Schwannoma (Figure 1). The treatment consisted of wide local excision and reconstruction. Intra-operatively, the tumor was found to infiltrate the periosteum and abutting the outer table of the occipital bone. A wide local excision of the tumor, the periosteum and the outer table of the bone was done (Figure 1). Histopathology confirmed malignant Schwannoma infiltrating into the outer table. The wound was initially managed by moist occlusive dressings in the interim till the histopathology report was available. One month after the initial surgery, the wound was closed with a large bipedicle bucket handle flap from the parieto-occiptal scalp region (Figure 1). There was some graft loss on the donor area and hence the patient was again scheduled for skin grafting of the donor area after two months (Figure 1). The postoperative course was unremarkable and without any complications. The patient was then referred to the Oncology unit for radiation therapy.

## DISCUSSION

The degeneration of neurofibromas is a rare complication of neurofibromatosis, and the lifetime risk for a patient of NF1 is of the order of 3 to 5 percent.^7^^-^^9^ Histologically, the neurofibrosarcoma is characterized by the presence of fusiform cells, probably derived from Schwann cells. Collagen fibers are rare. There is a presence of coarse reticulin fibrils, in parallel rows between fusiform cells which is very characteristic of the lesion. Signs of malignancy are represented by pleomorphic cells, giant cells, mono or polynuclear cells, an excess of mitoses, an invasion of surrounding tissues, and vascular invasion.^10^^,^^11^

The degeneration of a plexiform neuroﬁbrome must be considered specifically because there is usually a rapid increase in size and volume of the lesion. There is usually also presence of pain, induration and neurologic signs. 5-year survival rates range from 48 to 58%. Recurrence rates vary from 38 to 45 percent and overall survival at 10 years does not exceed 20 to 40%. Hence, frequent monitoring is essential.^12 ^The classic treatment of cephalic and scalp involvement of the malignant Schwannoma is not codified. It is variable from patient to patient, depending on the location and the level of involvement of structures. Whatever the histological grade, treatment consists of wide local excision, complete with radiotherapy for residual microscopic disease. First line chemotherapy can be tested to facilitate resection, however the rate of response is only 25-30 percent.^13^

The challenge in reconstructive surgery is to adapt processes to deal with the various problems posed by this disease.^13^ Surgical excision followed by suitable reconstruction is essential.^14^ The surgical reconstructive options are based on the extent of the disease and the resection margins, the viability of the surrounding local tissues, the local and the regional or microvascular flap options as well as the hemodynamic status of the patient. Neurofibromatosis is a common pathology, however degeneration of plexiform neurofibromas remains a serious and often underestimated complication. Prompt evaluation, wide surgical excision and reconstruction is essential to minimize mortality and morbidity.
